# Hederagenin potentiated cisplatin- and paclitaxel-mediated cytotoxicity by impairing autophagy in lung cancer cells

**DOI:** 10.1038/s41419-020-02880-5

**Published:** 2020-08-13

**Authors:** Kun Wang, Xiaodong Liu, Quanmeng Liu, Idy ht Ho, Xianli Wei, Ting Yin, Yujuan Zhan, Wenjing Zhang, Wenbo Zhang, Bonan Chen, Jiangyong Gu, Yuhui Tan, Lin Zhang, Matthew Tv Chan, William Kk Wu, Biaoyan Du, Jianyong Xiao

**Affiliations:** 1 Department of Pathology, Guangzhou University of Chinese Medicine, Guangzhou, 510006 China; 2 Research Center of Integrative Medicine, School of Basic Medical Sciences, Guangzhou University of Chinese Medicine, Guangzhou, 510006 China; 3 Department of Anaesthesia and Intensive Care, The Chinese University of Hong Kong, Hong Kong, 999077 China; 4 Department of Hand and Foot Surgery, Shandong Provincial Hospital affiliated to Shandong University, Shandong, 250100 China; 5 Department of Medical Instruments, Guangdong Food and Drug Vocational College, Guangzhou, 510520 Guangdong China; 6 Department of Biochemistry, Guangzhou University of Chinese Medicine, Guangzhou, 510006 China; 7 The Second Clinical College, Guangzhou University of Chinese Medicine, Guangzhou, 510006 China

**Keywords:** Lung cancer, Pharmacology

## Abstract

Autophagy inhibition has been demonstrated to increase the efficacy of conventional chemotherapy. In this study, we identified hederagenin, a triterpenoid derived from *Hedera helix*, as a potent inhibitor of autophagy and then hypothesized that hederagenin might synergize with chemotherapeutic drugs (e.g., cisplatin and paclitaxel) to kill lung cancer cells. Firstly, we observed that hederagenin induced the increased autophagosomes in lung cancer cells concomitantly with the upregulation of LC3-II and p62, which indicated the impairment of autophagic flux. The colocalization assay indicated hederagenin could not block the fusion of lysosomes and autophagosomes, whereas the lysosomal acidification might be inhibited by hederagenin as revealed by the reduced staining of acidity-sensitive reagents (i.e., Lysotracker and acridine orange). The aberrant acidic environment then impaired the function of lysosome, which was evidenced by the decrease of mature cathepsin B and cathepsin D. Lastly, hederagenin, in agree with our hypothesis, promoted pro-apoptotic effect of cisplatin and paclitaxel with the accumulation of reactive oxygen species (ROS); while the synergistic effect could be abolished by the ROS scavenger, N-acetyl-L-cysteine. These data summarily demonstrated hederagenin-induced accumulation of ROS by blocking autophagic flux potentiated the cytotoxicity of cisplatin and paclitaxel in lung cancer cells.

## Introduction

Lung cancer is the most common cancer and the leading cause of cancer death worldwide^1^. Although promising progress has been made in lung cancer treatment, the 5-year survival rate is still very low^2^. The development of new regimens or new anticancer strategies will undoubtedly provide great benefits to the patients and society.

Autophagy is a highly conserved mechanism for the degradation and recycles of cellular components, including macromolecules and damaged organelles. Dual roles of autophagy in the tumorigenesis and cancer progression have been widely studied and intensively reviewed^3–5^. On the one hand, autophagy may limit malignant transformation at the early stage of tumorigenesis. For instance, deficiencies of crucial autophagy genes, such as BECN1, may promote spontaneous development of lung cancer^6^. Conditional knockout of Atg5 or Atg7 in mice also accelerated KrasG12D- and BrafV600E-driven lung carcinomas, respectively^7,8^. On the other hand, high basal levels of autophagy could be observed in established tumors, especially when KRAS or BRAF mutations occurred^3^. In this context, autophagy induction promotes cancer progression and protects against cancer cell death by antagonizing the metabolic stress, hypoxia, and anoikis^3^. It is therefore plausible that inhibition of autophagy has been considered to be a novel anticancer strategy.

It has been widely reported that autophagy inhibition may enhance the anticancer activities of conventional chemo- and radiotherapeutic regimens^9,10^. For example, chloroquine (CQ) and its derivative hydroxychloroquine (HCQ) that are well-known late-phase autophagy inhibitors have been approved by the US Food and Drug Administration (FDA) for the treatment of malaria and rheumatoid arthritis. More than 80 clinical trials have been officially launched to investigate the therapeutic potentials of CQ (20 trials) and HCQ (60 trials) as single agents or as a part of combinatorial therapeutic regimens in the treatment of various cancer types, including lung cancer (http://www.clinicaltrials.gov/). While neither CQ nor HCQ alone provided significant antineoplastic benefits, they hold the potentials to improve the outcome of conventional therapeutic interventions^11^. In this regard, the identification of novel autophagy inhibitors may provide more options for agents for combinatorial therapy in the future.

Hederagenin is a triterpenoid isolated from the plant Hedera Helix (also known as the common Ivy). Recently, hederagenin was reported to exert anti-cancer^12^, anti-inflammatory^13^, anti-atherosclerosis^14^, and anti-depression^15^ activities in vitro and in vivo. In the present study, the effects of hederagenin on autophagy was investigated in the lung cancer cells. Its action as an adjuvant to sensitize the anticancer activities of cisplatin and paclitaxel was also explored.

## Materials and methods

### Chemicals

Hederagenin (A0423) and paclitaxel (A0177) were purchased from Chengdu Must Bio-Technology (Chengdu, China); Cisplatin (S1166), bafilomycin A_1_ (S1413), and N-acetyl-L-cysteine (NAC, S1623) were products of Selleck Chemicals (Houston, TX, USA). Cisplatin was dissolved in dimethylformamide, and other chemicals were dissolved in dimethyl sulfoxide (DMSO) upon receipt. All chemicals were aliquoted and stored at −80 °C.

### Cell culture

The human lung cancer cell lines NCI-H1299 (ATCC^®^ CRL-5803^™^) and NCI-H1975 (ATCC^®^ CRL-5908^™^) were obtained from American Type Culture Collection (ATCC). All the cells were maintained in Dulbecco’s Modified Eagle Medium supplemented with 10% (v/v) of heat-inactivated fetal bovine serum and 1% (v/v) of penicillin-streptomycin solution in a humidified atmosphere of 5% (v/v) CO2 and 95% (v/v) air. All cell culture-related reagent was obtained from Thermo Fisher Scientific (San Jose, CA, USA).

### Cell viability assay

NCI-H1299 or NCI-H1975 cells were seeded onto a 96-well plate in a density of 3000 cells/well and treated for 24 h. For determining drug synergism, NCI-H1299 cells were firstly treated with hederagenin (50 μM) for 4 h, followed by the administration of paclitaxel or cisplatin for 24 h. At the end of the assay, 10 µL of the Cell Counting Kit-8 (CCK8) solution was added to each well. Then the plate was incubated at the cell culture incubator for 2 h. The cell viability in each well was determined by optical density at 450 nm.

### Annexin V/propidium iodide (PI) apoptosis assay

Cells were seeded onto 6-well plates at a density of 2.5 × 10^5^ cells/well and assigned into four groups. In the single treatment group, cells were administered with hederagenin, paclitaxel, or cisplatin for 24 h before harvest. For the combinatorial treatment group, cells were treated with hederagenin for 4 h, followed by paclitaxel or cisplatin for another 24 h. Cells were then trypsinized and harvested for staining using Annexin V-FITC/PI, according to manufacturer’s instructions (556547, BD Pharmingen, San Diego, CA, USA). Apoptotic cells were counted by flow cytometry (BD Pharmingen, San Diego, CA, USA). FITC^+^/PI^-^ fraction and FITC^+^/PI^+^ fraction was considered as apoptotic cells.

### Western blotting

Total protein was extracted with cell lysis buffer (50 mM Tris, 150 mM NaCl, 1% (v/v) NP-40, 1 mM EDTA, pH7.6) containing a cocktail of protease inhibitors. Samples (30 µg protein/lane) were separated on SDS-polyacrylamide gels, then transferred onto PVDF membranes (0.22 µm pore, Roche). After blocking with TBST buffer (20 mM Tris, 137 mM NaCl, 0.1% Tween-20, pH8.0) containing 5% (w/v) nonfat milk, membranes were incubated with primary antibodies against β-actin (1:1500; 3700), LC3B (1:1500; 3868), p62 (1:1500; 88588), Cathepsin B (1:1500; 31718), Cathepsin D (1:1500; 2284), caspase-3 (1:1500; 9662), or cleaved-poly (ADP-ribose) polymerase (PARP) (1:1500; 5625) overnight at 4 °C. Then membranes were incubated with secondary antibody (1:4000) for 1 h at room temperature. These primary antibodies were obtained from Cell Signaling Technology (Boston, MA, USA). The secondary antibodies, including peroxidase-labeled anti-mouse IgG (AS004) and anti-rabbit IgG (AS014), were ordered from Abclonal (Wuhan, China). The protein bands were visualized using Immobilon Western Chemiluminescent HRP substrate (WBLS0500, Millipore, Burlington, MA, US).

### Transfection

pBABEpuro GFP-LC3 (Addgene plasmid #22405) and pBABE-puro mCherry-GFP-LC3B (Addgene plasmid #22418) were gifts from Jayanta Debnath^16,17^. Cells were seeded onto glass coverslips in 12-well plates and transiently transfected with 0.5 µg of plasmids using the Lipofectamine 3000 kit according to the manufacturer’s instructions (Thermo Scientific, San Jose, CA, USA). After 24 h, cells were treated accordingly, fixed with 4% (w/v) paraformaldehyde (PFA), and then visualized with an LSM 800 confocal microscope (Carl Zeiss, Jena, Germany). The number of puncta per cell was counted and compared by researchers who were blinded for grouping information.

### LysoTracker and acridine orange staining

LysoTracker^TM^ Red DND-99 (LysoTracker) and acridine orange (AO) were obtained from Thermo Fisher Scientific (San Jose, CA, USA). After treatment, cells were labeled with LysoTracker (75 nM) or AO (1 µg/mL) for 15 min at 37 °C. Cells were then washed three times with PBS and fluorescence was visualized under a confocal microscope (LSM 800, Carl Zeiss, Jena, Germany).

### Lysosomal pH measurement

Quantification of lysosomal pH was performed using a ratiometric lysosomal pH dye LysoSensor^™^ Green DND-189 (Thermo Scientific, San Jose, CA, USA). Cells were seeded on the 35 mm confocal dishes and assigned into calibration curve groups (pH = 4.5, 5, 5.5, 6, 6.5, 7) or experimental groups. After treatment, cells were labeled with 1 µM LysoSensor^™^ Green DND-189 for 1 h at 37 °C in regular medium. Excessive dye was then washed out using PBS. For experimental groups, cells are ready for fluorescence measurements under a confocal microscope (LSM 800, Carl Zeiss, Jena, Germany). For calibration curve groups, cells were treated for 10 min with 10 µM monensin and 10 µM nigericin in MES buffer with various pH from 4.5 to 7. The MES buffer consisted of 5 mM glucose, 20 mM MES, 1 mM CaCI_2_, 1 mM MgCI_2_, 130 mM NaCl, and 10 mM KCI. The pH of the MES buffer was adjusted by NaOH or HCl. At least 40 cells from each group were imaged by a confocal microscope. The integrated density of pH-dependent fluorescence was measured by Image J software. The pH calibration curve was generated from the plot using Microsoft Excel. The pH of the experimental groups was calculated according to the fluorescence density and calibration curve.

### Transmission electron microscopy

NCI-H1299 cells were seeded onto 10 cm dishes and cultured until they reached 50% confluence. After drugs treatment for 24 h, cells were scraped from the dishes and centrifugated to obtain cell pellets, following by fixation with 2.5% glutaraldehyde solution for overnight. The fixed pellets were then washed with 0.1 M phosphate buffer, further fixed with 1% osmic acid for 3 h, dehydrated with alcohol, and propylene oxide solution. Then cell pellets were soaked and embedded in Spurr resin, followed by polymerizing in an oven set at 70 °C. Finally, ultrathin sections were cut, and then stained with lead citrate and uranyl acetate. Cell organelles were visualized under a transmission electron microscope (#HT7700, Hitachi, Tokyo, Japan).

### Reactive oxygen species detection

Cells were seeded and treated as the Annexin V/PI assay. 2′,7′-dichlorodihydrofluorescein diacetate (H2DCFDA, D399, Thermo Fisher Scientific, San Jose, CA, USA) was applied to detect the intracellular reactive oxygen species (ROS) levels. After treatment, cells were trypsinized and labeled with H2DCFDA (10 µM) for 20 min at 37 °C. Cells were then washed three times with PBS and analyzed by flow cytometry.

### Human lung cancer xenografting

All the animal experiments were approved by the Animal Ethics Committee at Guangzhou University of Chinese Medicine (Approval No. 20200526012). Five-week-old BALB/c nude mice with body weights ranging from 18 to 22 g were obtained from Guangdong Medical Laboratory Animal Center. The nude mice were housed in the specified-pathogen-free animal laboratory. They were given access to sterilized food and water properly. After a 1-week acclimation period, NCI-H1299 cells (6 × 10^6^ in 200 µL) were subcutaneously injected into the right flanks of the mice. The mice were examined every day. When the tumors reached a diameter of 0.1 mm^3^, the nontumor mice were excluded and the remain were randomly assigned into four groups (*n* = 8 per group) that were daily treated with vehicle (2% DMSO, 30% PEG300, and 3% tween-80 in saline solution), hederagenin (25 mg/kg), cisplatin (1 mg/kg), or a combination of hederagenin and cisplatin. The investigator was blinded to the group allocation during the experiment. Tumor volume was measured every day after grouping. The mice were sacrificed on the 11th day after drug treatment. The tumors were then dissected and weighed.

### Statistical analysis

All the conclusions were made based on three independent experiments. The investigator was blinded to the group allocation during when assessing the outcome. The sample size was chosen to ensure adequate power to detect a pre-specified effect size according to the previous reports^18,19^. The data meet the assumptions of normal distribution and were presented as mean ± S.D. (standard deviation of the mean). One-way analysis of variance (ANOVA) and multiple comparisons were applied to analyze the differences among groups. If the variance is similar between the groups, LSD (Least-significant difference) method is chosen for comparison, otherwise the Games-Howell method is required. A *p* < 0.05 was considered to be of statistical significance. For drug synergism studies, the *Q* value was derived as follows: Eab/(Ea + Eb − Ea × Eb), where Ea and Eb represent the effects of drugs a and b, respectively, and Eab represents the combined effect. A *Q* value >1.15 indicates a synergistic relationship. A *Q* value between 0.85 and 1.15 indicates an additive relationship; a *Q* value of <0.85 indicates that a and b are antagonistic^20^.

## Results

### Hederagenin inhibited autophagy flux in lung cancer cells

**Fig. 1:**
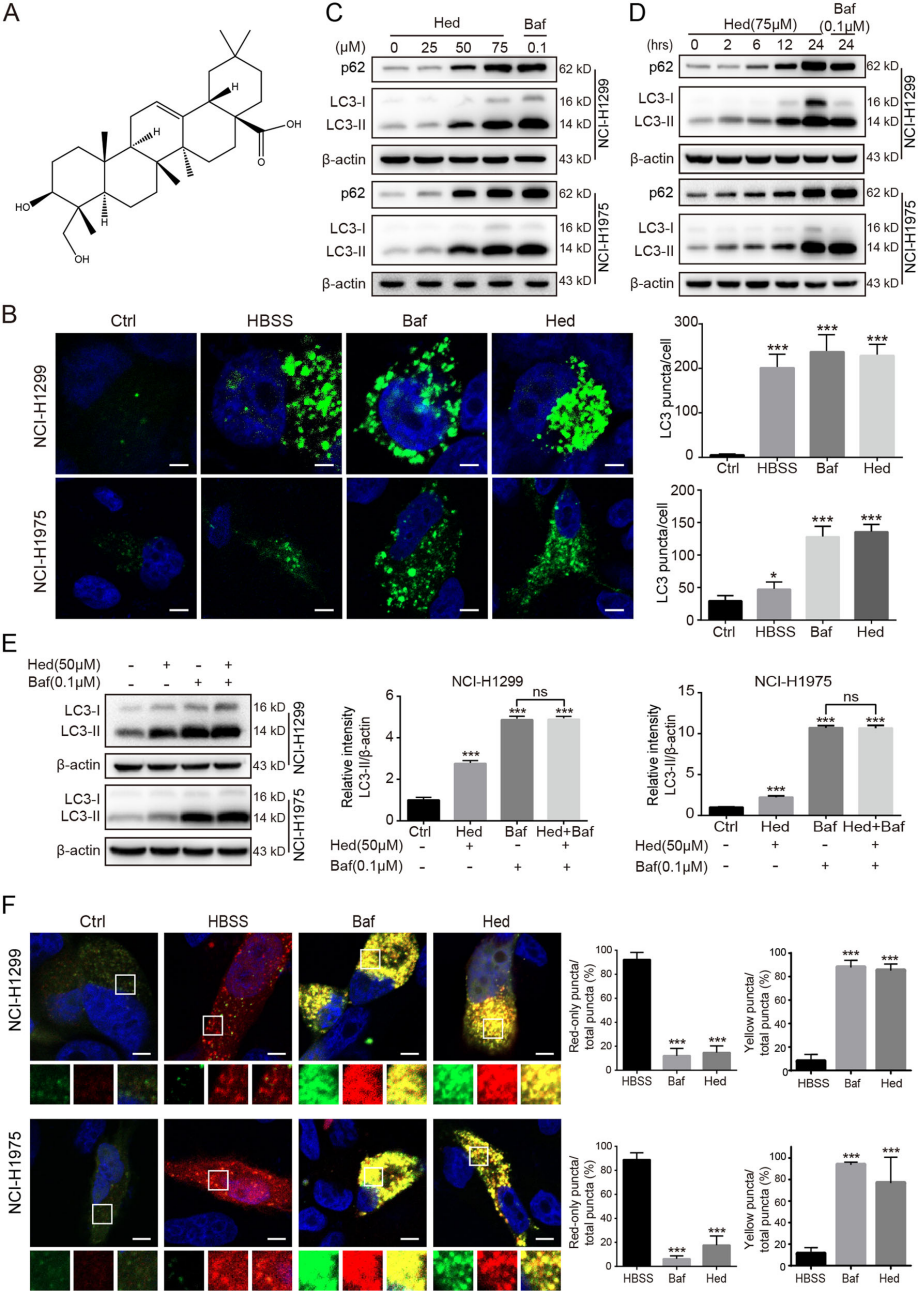
Hederagenin inhibited autophagy flux in lung cancer cells. **a** Chemical structure of hederagenin was shown. **b** HBSS, bafilomycin A_1_ and hederagenin induced dramatic accumulation of LC3-GFP puncta. The bar chart showed the number of LC3 positive puncta per cell (*n* = 15). Scale bar, 5 μm. **c** Hederagenin caused p62 accumulation and LC3-I to LC3-II conversion in a dose-dependent manner. **d** Hederagenin caused p62 accumulation and LC3-I to LC3-II conversion in a time-dependent manner. **e** Co-treatment with hederagenin failed to further increase bafilomycin A_1_ induced LC3-II accumulation. **f** Cell starvation by HBSS treatment triggered a large number of mCherry puncta along with only a few GFP-puncta. Both GFP and mCherry puncta were accumulated after bafilomycin A_1_ or hederagenin administration. Comparing to HBSS, the colocalization of red and green fluorescence was significantly higher in bafilomycin A_1_ or hederagenin treatment group. Twenty cells were randomly selected from each group, the number of red-only, yellow or total puncta was counted. ***p < 0.001, ANOVA with multiple comparisons. Scale bar, 5 μm. Hederagenin: Hed; bafilomycin A_1_: Baf.

The chemical structure of hederagenin was shown in Fig. 1A. Initially, we conducted a pilot experiment to observe the effects of hederagenin on autophagy of lung cancer cells. Two lung cancer cell lines (i.e., NCI-H1299 and NCI-H1975) characterized by high metastasis were cultured and transfected with LC3-GFP plasmid. Upon the treatment of hederagenin (50 μM), the number of LC3-positive puncta was dramatically increased in both cells, similar to the HBSS starvation group (induction of autophagy) and bafilomycin A_1_ (autophagy inhibitor) treatment (Fig. 1B). As LC3 is a typical marker of the autophagosome, the preliminary data indicated that hederagenin increased the number of autophagosomes in lung cancer cells. The results were confirmed by western blot analysis that showed hederagenin treatment caused the upregulation of LC3-II in a time- and dose-dependent fashion (Fig. 1C, D).

The increased autophagosomes theoretically may result from the promoted initiation of autophagy or the inhibition of autophagic degradation. Thus, we examined the level of p62 by western blot that showed a time- and dose-dependent upregulation. Because p62 was mainly degraded by the lysosome-dependent autophagic pathway, the level of p62 inversely correlated with autophagic activity. The upregulation of LC3-II and p62 suggested hederagenin interfered with the autophagic degradation, i.e., blocked the late autophagic flux. However, the data cannot exclude the possibility the early stage of autophagic flux (i.e., the initiation of autophagy) may simultaneously be upregulated. To further monitor the process of the autophagic flux, the lung cancer cells were treated with hederagenin or/and bafilomycin A_1_ followed by the examination of LC3-II level (LC3 turnover assay). Bafilomycin A_1_ can inhibit lysosomal acidification, thereby blocking the degradation of LC3-II. In the presence of bafilomycin A_1_, hederagenin failed to increase LC3-II level in both cell lines (Fig. 1E), suggesting that hederagenin functioned similarly with bafilomycin A_1_ to block late autophagic flux rather than to promote the initiation of autophagy. We also detected the effects of hederagenin on autophagy in several other types of cancer cells. Consistently, hederagenin administration also led to elevation of LC3-II and p62 levels in A375 human melanoma, CNE2 human nasopharyngeal carcinoma, HepG2 human liver cancer, and MGC803 human gastric cancer cell lines, indicative of a common autophagy inhibition in cancer cells derived from different tissues (Fig. S1).

### Hederagenin impaired autophagic flux by inhibiting lysosomal acidification

**Fig. 2:**
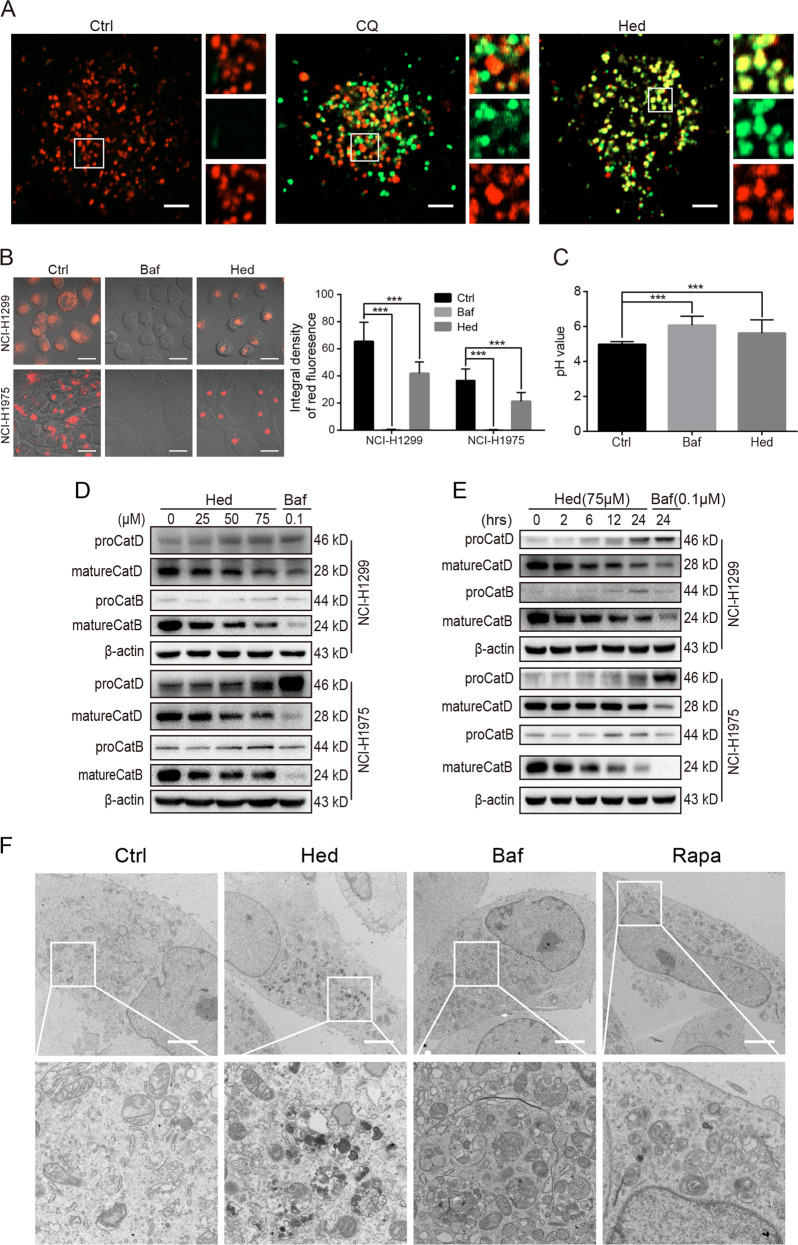
Hederagenin impaired autophagic flux by inhibiting lysosomal acidification. **a** NCI-H1299 cells stably expressing GFP-LC3, a canonical autophagosome marker with green fluorescence, were treated with vehicle, chloroquine (CQ, 20 µM), or hederagenin (50 µM) for 24 h. LysoBrite^TM^ red dyes were used to label the lysosomes. Images were acquired with a confocal laser scanning microscope. Typical images are shown. Scale bar, 5 μm. **b** Acidic environment-dependent LysoTracker fluorescence was greatly suppressed by hederagenin and bafilomycin A_1_ treatment. Scale bar, 20 μm. **c** Lysosomal pH was detected by LysoSensor^™^ Green DND-189 in NCI-H1299 cells (*n* = 40 cells/group). Cells were treated with hederagenin (50 µM) or bafilomycin A_1_ (0.1 µM) for 6 h. Similar to the effect of bafilomycin A_1_ (inhibitor of V-ATPase), hederagenin treatment significantly increased the lysosomal pH compared with the control. ***p < 0.001, ANOVA with multiple comparisons. **d** & **e** Hederagenin disturbed CatB and CatD processing in a dose- and time-dependent manner. Hederagenin: Hed, bafilomycin A_1_: Baf, rapamycin: Rapa. **F** Hederagenin induced excessive accumulation of undigested cargos (Transmission electron microscopy). NCI-H1299 cells were treated with hederagenin (50 µM), rapamycin (500 nM), or bafilomycin A1 (100 nM) for 24 h. Then transmission electron microscopy was performed to observe the structure of the cells. Scale bar, 3 μm.

To reveal the potential mechanism by which hederagenin blocked the late autophagic flux, we also transfected lung cancer cells with the mCherry-GFP-LC3B tandem construct that can label autophagosomes with yellow fluorescence (overlap of mCherry^+^ and GFP^+^) and autolysosomes with red (quenching of GFP signal due to low pH inside lysosome). Similar to bafilomycin A_1_, Hederagenin (50 μM) treatment for 24 h caused substantial increases in the number of yellow puncta in NCI-H1299 and NCI-H1975 cells (Fig. 1F), indicating the accumulation of autophagosomes or non-acidified autolysosomes. In contrast, the HBSS starvation group mainly caused increases in red (i.e., mCherry only) signals. These data suggested that hederagenin might inhibit the delivery of mCherry-GFP-LC3 into lysosome or impair the lysosomal acid environment. To clarify whether hederagenin interfered with autophagosome-lysosome fusion or inhibited the acidification of lysosome, NCI-H1299 cells were transfected with GFP-LC3 as a marker of autophagosomes and subsequently treated with DMSO (vehicle), CQ, or hederagenin. The lysosomes were labeled by LysoBrite^TM^ red. In the vehicle-treated cells, signals for lysosomes (red) but not GFP-LC3 puncta, were present. CQ, a well-known autophagy inhibitor, was used to interfere with autophagosome-lysosome fusion. As expected, CQ treatment induced the accumulation of GFP-LC3 puncta that were only partially colocalized with the lysosomes. In contrast, hederagenin administration resulted in a substantial co-localization of LC3 and lysosome signals alone with a few red or green-only puncta (Fig. 2A). These results suggested that hederagenin was unlikely to affect the fusion of autophagosomes and lysosomes.

Then we asked if hederagenin inhibited the acidification of lysosomes. LysoTracker-stained cells showed that treatment with hederagenin significantly attenuated acid-dependent red fluorescence as compared with vehicle control (Fig. 2B). Instead, bafilomycin A_1_ as a positive control known to block lysosome acidification through inhibiting vacuolar-type H^+^-ATPase (V-ATPase) reduced LysoTracker fluorescence signals (Figs. 2B and S2). Similar results were observed with the staining of AO, which emitted bright red fluorescence in acidic vesicles (Fig. S2). Furthermore, the lysosomal pH was measured in NCI-H1299 cells after treatment with hederagenin or bafilomycin A_1_. As expected, bafilomycin A_1_ increased lysosomal pH by 6.07 ± 0.52 in NCI-H1299 cells. Although the effect of hederagenin on acidification of lysosome did not reach the extent that bafilomycin A1 did, cells treated with hederagenin had a significantly higher lysosomal pH than control cells (5.61 ± 0.77 vs. 4.97 ± 0.16, *p* < 0.001, Fig. 2C). The sustained acidic environment of the lysosome is essential for the maturation of internal pro-formed hydrolases, and then we examined the mature form of the representative Cathepsin B and D. As demonstrated in Fig. 2D, E, hederagenin treatment reduced levels of the mature form of cathepsin B and cathepsin D in a dose- and time-dependent manner. These results revealed that hederagenin inhibited autophagic flux by suppressing lysosomal acidification but not interfering with autophagosome-lysosome fusion. To further confirm the blockage of autophagic flux by hederagenin, the cell ultrastructure was visualized with transmission electron microscope. As demonstrated in Fig. 2F, hederagenin treatment caused a number of single membrane-bound vesicles containing deeply stained organelles-like structure in cells, which can also be identified in cells treated with bafilomycin A1, the well-accepted autophagic flux inhibitor.

### Hederagenin enhanced the anticancer effects of cisplatin

Chemotherapy is one of the main strategies to treat lung cancer, and its main anticancer mechanism is to induce apoptosis by torturing mitochondria. However, cancer cells can avoid the accumulation of death signals by activating the autophagy pathway to clear damaged mitochondria^3^. Therefore, the upregulation of autophagy was recognized as one of the important mechanisms leading to chemoresistance.

**Fig. 3:**
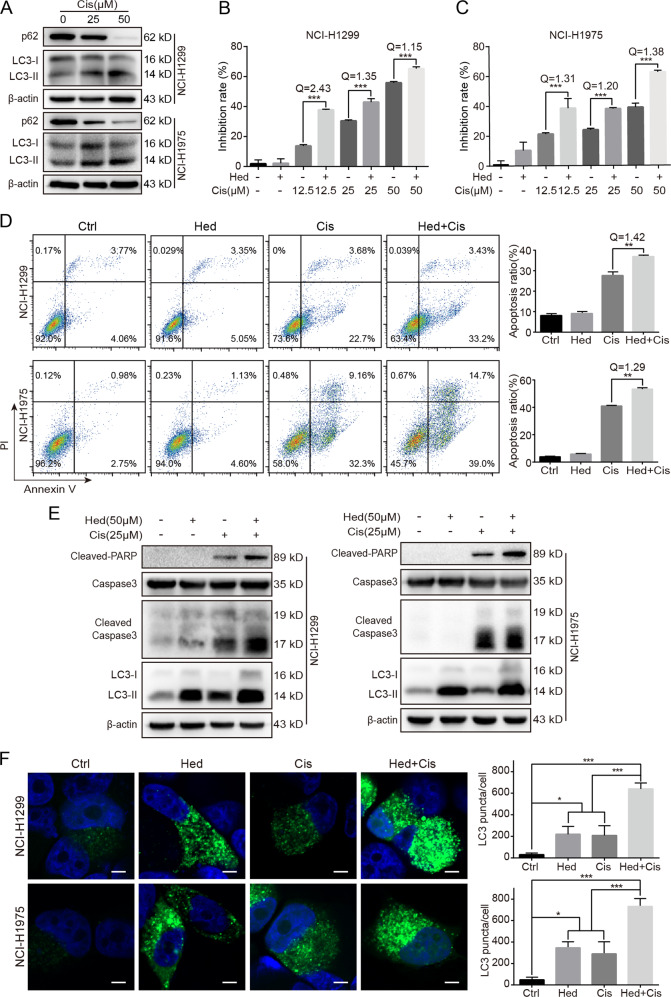
Hederagenin enhanced the anticancer actions of Cisplatin. **a** Cisplatin-induced autophagy as indicated by increased LC3-I to -II conversion and decreased p62 level. **b** & **c** Treatment with hederagenin (50 μM) showed no or little cytotoxicity to NCI-H1299 or NCI-H1975 cells. Pre-treatment with hederagenin (50 μM) enhanced Cis-mediated inhibition at all concentrations tested (12.5, 25, and 50 μM). ***p < 0.001, ANOVA with multiple comparisons. **d** Co-treatment with hederagenin and Cis induced significantly higher apoptosis than Cis alone. **p < 0.01, ANOVA with multiple comparisons. **e** Pre-treatment with hederagenin promoted Cis-triggered Caspase-3 and PARP cleavage. Compared with a single reagent, the LC3-II level was further upregulated when the combination of hederagenin and cisplatin was administrated. **f** Hederagenin and cisplatin co-treatment resulted in significant increases of GFP-LC3 puncta compared with a single reagent treatment group (*n* = 20 cells/group). *p < 0.05, **p < 0.01,***p < 0.001, ANOVA with multiple comparisons. Scale bar, 5 μm. Cisplatin: Cis, hederagenin: Hed.

In our pilot experiment, cisplatin treatment resulted in the accumulation of LC3-II and reduction of p62 in both NCI-H1299 and NCI-H1975 cells, suggesting that autophagy was upregulated (Fig. 3A). In theory, inhibition of autophagy may promote the anticancer effects of chemotherapeutic drugs. Several publications suggested that typical autophagy inhibitors (such as bafilomycin A_1_ and CQ) could promote chemotherapy-induced apoptosis in cancer cells. Then we asked whether hederagenin could also synergize with chemotherapy drugs to inhibit lung cancer. Our results showed that hederagenin strongly inhibited autophagy at the concentration of 50 μM. With the same concentration, hederagenin by itself failed to inhibit cell proliferation as indicated by CCK8 assay (Fig. 3B, C). However, when co-treated with cisplatin at different concentrations, hederagenin significantly enhanced the cytotoxic effects (Fig. 3B, C). Similar synergistic effects were also observed in apoptosis assay. In NCI-H1299 cells, treatment with cisplatin for 24 h remarkably induced early apoptosis (27.84 ± 1.79%, Annexin V^+^/PI^-^). Whereas hederagenin per se failed to cause any apoptosis, the combination of hederagenin and cisplatin significantly enhanced apoptotic cell death as compared with cisplatin alone (37.16 ± 0.61% vs. 27.84 ± 1.79%, *p* < 0.01, Fig. 3D). In NCI-H1975 cells, cisplatin administration induced both early and late (Annexin V^+^/PI^+^) apoptosis. Similarly, hederagenin co-treatment led to significantly more substantial apoptotic cell death than cisplatin alone (53.37 ± 0.95% vs. 41.03 ± 0.36%, *p* < 0.01, Fig. 3D). These synergistic effects on apoptosis were further confirmed by Western blot assay. As shown in Fig. 3E, cisplatin alone was sufficient to increase the level of cleaved caspase-3 and PARP. Co-treatment with Hed caused further accumulation of these apoptotic markers (Fig. 3E). Moreover, the combinatorial treatment of hederagenin and cisplatin resulted in significant increases of LC3-II protein level (Fig. 3E) and GFP-LC3 puncta (Fig. 3F) compared with single reagent treatment. These data suggested that the synergizing effects of hederagenin on cisplatin mediated cytotoxicity were related to the regulation of autophagy.

### Hederagenin promoted the anticancer effects of paclitaxel

**Fig. 4:**
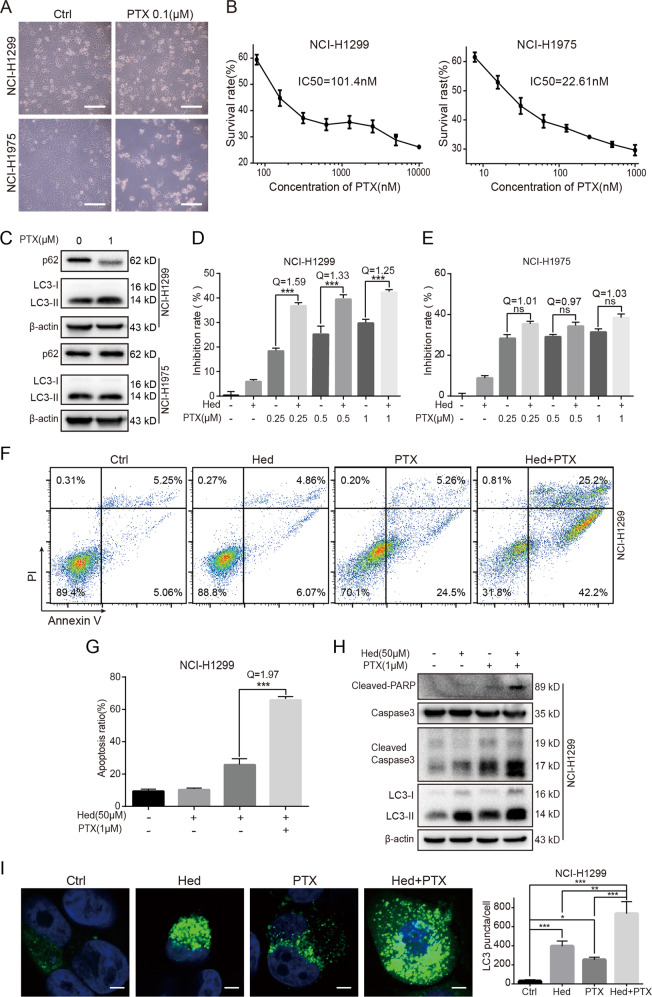
Hederagenin promoted the anticancer actions of paclitaxel. **a** NCI-H1299 cells and NCI-H1975 responded to paclitaxel differently under the light microscopy. Scale bar, 50 μm. **b** NCI-1975 cells were sensitive to paclitaxel treatment. The IC50 for and NCI-H1299 cells and NCI-H1975 cells were 101.4 nM and 22.61 nM, respectively. **c** Paclitaxel treatment induced autophagy in NCI-H1299 cells but not in NCI-H1975 cells as indicated by LC3 immunoblotting. **d** & **e** Hederagenin significantly synergized paclitaxel-induced growth inhibition in NCI-H1299 cells, but not in NCI-H1975 cells. **f** & **g** A combination of hederagenin and paclitaxel caused a significantly higher apoptosis rate in NCI-H1299 cells with a *Q* value of 1.97. ***p < 0.001, ANOVA with multiple comparisons. **h** Pre-treatment with hederagenin promoted paclitaxel-triggered Caspase-3 and PARP cleavage. Compared with single reagent treatment, the LC3-II level was further upregulated by hederagenin and paclitaxel co-treatment. **I** A combination of hederagenin and paclitaxel resulted in a significantly higher number of GFP-LC3 puncta than a single drug treatment group (*n* = 20 cells/group). *p < 0.05, **p < 0.01,***p < 0.001, ANOVA with multiple comparisons. Scale bar, 5 μm. Paclitaxel: PTX, hederagenin: Hed.

Paclitaxel is another first-line chemotherapeutic drug for lung cancer. In the current study, we found that NCI-H1975 and NCI-H1299 exhibited differential responses to paclitaxel (Fig. 4A). CCK8 assay demonstrated that the half-maximal inhibitory concentration (IC50) of paclitaxel was 22.61 nM and 101.4 nM for NCI-H1975 and NCI-H1299 cells, respectively (Fig. 4B). Interestingly, paclitaxel treatment strongly induced autophagy in NCI-H1299 cells and to a much lesser extent in NCI-H1975 cells as revealed by increased LC3-II-to-LC3-I ratio and reduced p62 levels (Fig. 4C). This agreed with previous reports that autophagy might be induced in response to chemotherapy. The sensitization effects of hederagenin on paclitaxel treatment were therefore explored. Using CCK8 assay, we showed that co-treatment of hederagenin and paclitaxel caused significantly higher cytotoxicity than paclitaxel alone in NCI-H1299 cells (Fig. 4D). However, the synergizing effects of hederagenin were not evident in the NCI-H1975 cells (Fig. 4E). Likewise, apoptosis assay showed that hederagenin enhanced paclitaxel-induced early and late apoptosis in NCI-H1299 cells, compared to the paclitaxel-alone group (Fig. 4F, G). Finally, Western blots further demonstrated that hederagenin increased the levels of cleaved caspase-3 and PARP (Fig. 4H). Consistent with the findings of the treatment of cisplatin combined with hederagenin, pre-treatment with hederagenin also significantly increased paclitaxel-induced LC3-II protein level (Fig. 4H) and LC3 puncta (Fig. 4I) in NCI-H1299 cells. Taken together, these findings revealed that hederagenin might enhance the sensitivity to paclitaxel preferentially in cells with high autophagy induction, suggesting that autophagy inhibition contributed to the synergizing effects of hederagenin.

### Hederagenin sensitized lung cancer cells to cisplatin and paclitaxel by amplifying ROS stress

**Fig. 5:**
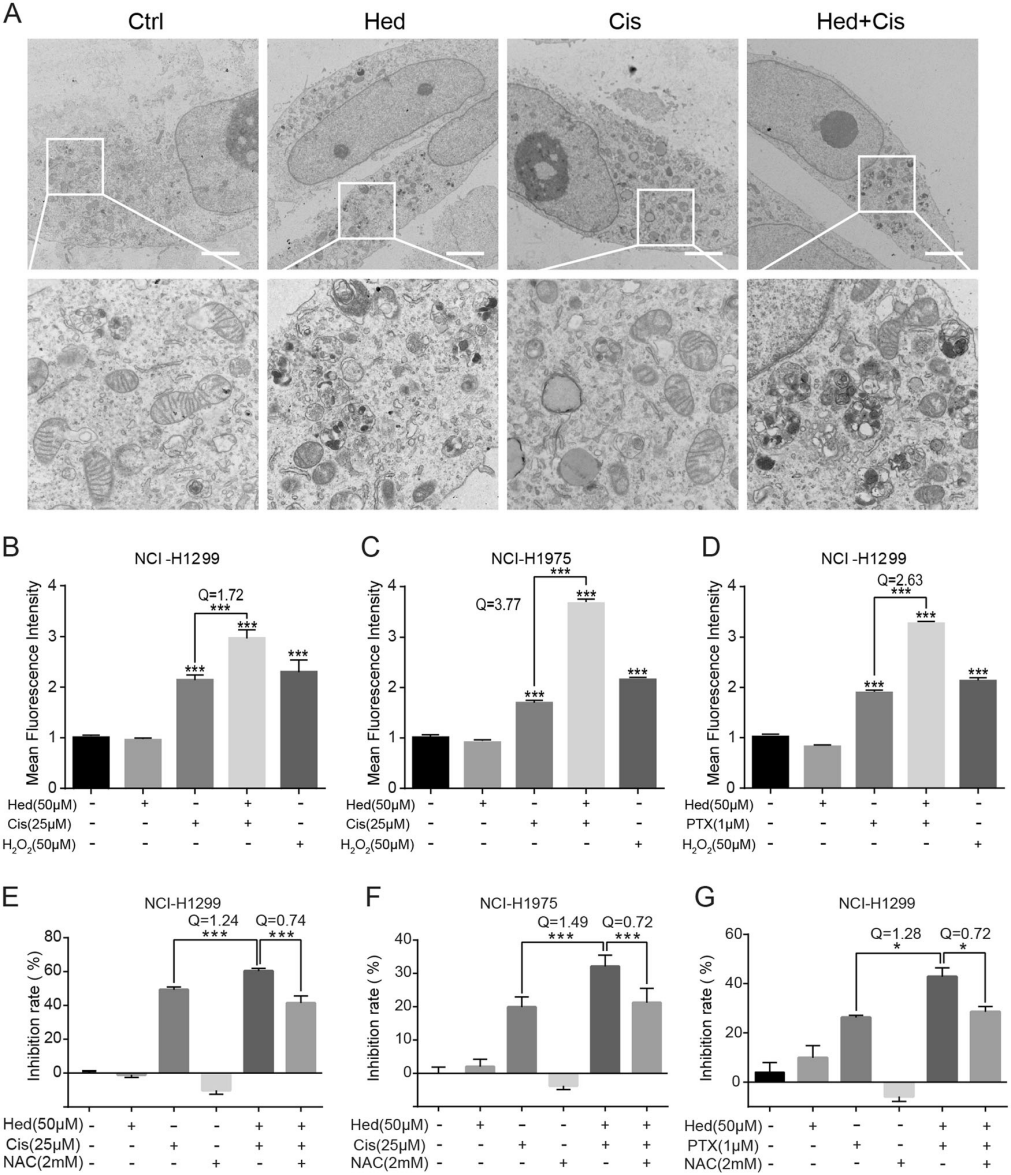
Hederagenin sensitized cancer cells to cisplatin and paclitaxel by amplifying ROS stress. **a** Hederagenin and cisplatin combination induced excessive accumulation of undigested cargos (Transmission electron microscopy). NCI-H1299 cells were treated with either hederagenin (50 µM), cisplatin (25 µM), or both for 24 h. Then transmission electron microscopy was performed to observe the structure of the cells. Scale bar, 3 μm. **b & c** Compared to cisplatin (25 μM) treatment alone, pre-treatment with hederagenin (50 μM) significantly enhanced Cis-induced ROS production in NCI-H1299 (*Q* = 1.72) and NCI-1975 cells (*Q* = 3.77). Cells treated by H_2_O_2_ (50 μM) for 2 h was used as a positive control. **d** Co-treatment with hederagenin (50 μM) and paclitaxel (1 μM) induced significantly higher intracellular ROS level than paclitaxel (1 μM) treatment alone in NCI-H1299 cells (*Q* = 2.63). Cells treated by H_2_O_2_ (50 μM) for 2 h was used as a positive control. **e & f** Pre-treatment with hederagenin (50 μM) synergized Cis (25 μM)-induced growth inhibition in NCI-H1299 (*Q* = 1.24) and NCI-H1975 (*Q* = 1.49) cells. These effects were completely abolished by NAC (2 mM) treatment. The *Q* value between the Hed + Cis group and the NAC + Cis + Hed group is 0.74. **g** Pre-treatment with hederagenin (50 μM) synergized paclitaxel (1 μM)-induced growth inhibition in NCI-H1299 cells (*Q* = 1.28). The synergizing effect was completely reversed by NAC (2 mM) pre-treatment. The *Q* value between the Hed + PTX group and the NAC + PTX + Hed group is 0.72. **p < 0.01, ***p < 0.001, ANOVA with multiple comparisons. Cisplatin: Cis, paclitaxel: PTX, hederagenin: Hed.

Previous studies revealed that ROS was crucial for apoptosis induction by chemotherapeutic drugs^21,22^. However, autophagy may prevent apoptosis in cancer cells by eliminating ROS or the damaged mitochondria which was a major source of ROS. Therefore, we asked whether hederagenin might restrain such a protective mechanism. Under the electron microscopy, considerable undigested organelles were observed in cancer cells treated with hederagenin, due to the blockage of autophagic flux (Fig. 2F and Fig. 5A). In the presence of cisplatin, which may cause mitochondria damage and lethal level of ROS, organelles-like structures further accrue to cells after hederagenin treatment (Fig. 5A). This outcome suggested that due to hederagenin mediated autophagy inhibition, cells cannot clean deadly “waste” caused by cisplatin. Intracellular ROS levels were then determined by flow cytometry. Consistent with previous reports, cisplatin treatment caused moderate increases of ROS in lung cancer cells^23^. When applied separately, hederagenin did not affect the intracellular ROS levels (Fig. 5B-D). However, combination of hederagenin with cisplatin led to significantly higher ROS levels than cisplatin alone in NCI-H1299 cells (2.96 ± 0.17 vs. 2.13 ± 0.10, *p* < 0.001, Fig. 5B) and NCI-H1975 cells (3.66 ± 0.06 vs. 1.69 ± 0.04, *p* < 0.001, Fig. 5C). Similar responses were observed in paclitaxel treatment. Comparing to paclitaxel alone group, hederagenin significantly raised paclitaxel-induced ROS levels in NCI-H1299 cells (3.25 ± 0.01 vs. 1.88 ± 0.03, *p* < 0.001, Fig. 5D). These results suggested that ROS might play a role in the synergizing effects of hederagenin. To test this hypothesis, cells were pre-treated with NAC, a commonly used antioxidant to reduce ROS. Results indicated that NAC abolished the synergizing effects of co-treatment (Fig. 5E-G). Thus, we concluded that hederagenin-mediated inhibition of autophagy might elevate ROS levels, leading to the synergizing anticancer actions.

### The combination of hederagenin and cisplatin synergistically inhibited tumor growth in a mouse xenograft model

**Fig. 6:**
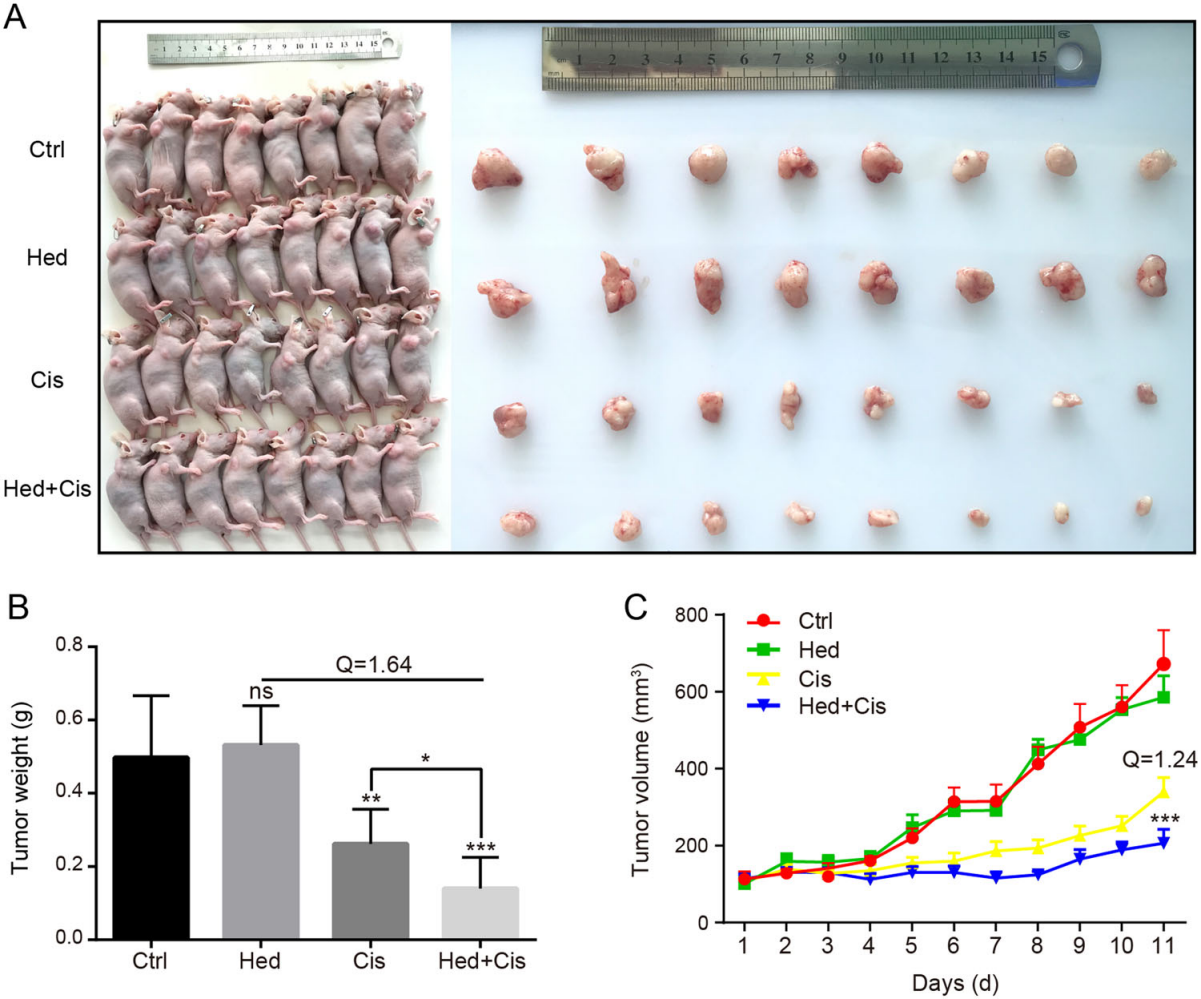
The combination of hederagenin and cisplatin inhibits tumor growth in an NCI-H1299 mouse xenograft model. **a** Xenograft tumors were induced in nude mice by subcutaneous injection of NCI-H1299 cells in the right flank. When the tumor volume reached 0.1 mm^3^, the mice were divided into four groups (*n* = 8 for each group) and treated by hederagenin (25 mg/kg) or cisplatin (1 mg/kg) or a combination of hederagenin and cisplatin. **b** The tumors were resected and weighed on the 11th day of drug treatment; data was visualized by GraphPad software. Error bars represent means ± SD. *p < 0.05, **p < 0.01, ***p < 0.001. *Q* value > 1.15 indicted a synergistic effect. **c** Tumor volume was measured and recorded every day and was lower in the combination-treated groups than in the cisplatin group. Data are presented as mean ± standard error. ***p < 0.001, Cis group vs. Hed+Cis group. *Q* value > 1.15 indicted a synergistic effect. Hederagenin, Hed; cisplatin, Cis.

To explore if the findings that hederagenin sensitized cancer cells to cisplatin-induced apoptosis could be replicated in vivo, we examined the effect of hederagenin on the therapeutic efficacy of cisplatin using a mouse xenograft model. NCI-H1299 cells were subcutaneously injected into the right flank of nude mice, followed by receiving vehicle, hederagenin (25 mg/kg), cisplatin (1 mg/kg) or a combination of hederagenin and cisplatin for 11 days starting about 1 week after tumor inoculation. The tumor sizes in the combination group was visually smaller than those in the other groups (Fig. 6A). The tumor weights were measured. As shown in Fig. 6B, the tumor weights in the combination group were significantly smaller than those in the cisplatin alone group (*p* < 0.05). The *Q* value was larger than 1.15, indicative of a synergistic effect of hederagenin and cisplatin. We also measured the tumor volumes every day. Hederagenin alone had no impact on the growth of tumors. While cisplatin expectedly reduced the growth rate of xenografted tumors, a combination of hederagenin with cisplatin further retard the tumor growth. These results suggested that the combination of hederagenin and cisplatin can synergistically inhibit tumor growth in vivo, which was consistent with the in vitro studies.

## Discussion

**Fig. 7:**
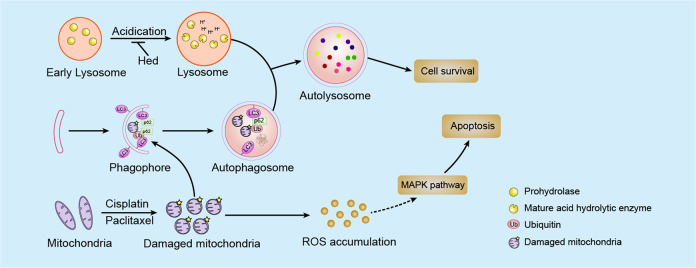
The proposed mechanisms of hederagenin-inhibited autophagy and the synergistic effects of hederagenin and chemotherapeutic agents. Hederagenin acted as a novel late-phase autophagy inhibitor by targeting lysosomes, and that hederagenin may synergize the cytotoxicity effects of cisplatin and paclitaxel by blocking the cell death preventive autophagy and amplifying cytotoxic ROS.

In summary (Fig. 7), we reported that hederagenin functioned as a potent novel autophagy inhibitor by inhibiting lysosomal acidification. As autophagy is considered to be an important mechanism of drug resistance^9,10^, then we questioned whether blockade of autophagy by hederagenin could promote the killing effect of cisplatin and paclitaxel. In agree with the speculation, pre-treatment with hederagenin synergized with cisplatin and paclitaxel to produce cytotoxicity in lung cancer cells. Such action was mediated through elevating intracellular ROS levels. Hederagenin and cisplatin showed a synergistic killing effect on both NCI-H1299 and NCI-H1975 cells. However, unlike cisplatin, the synergizing effects of hederagenin on paclitaxel treatment could only be observed in NCI-H1299 cells. These coincided with the phenomena that cisplatin treatment led to upregulation of autophagy in both cell lines, yet paclitaxel induced autophagy in NCI-H1299 cells more significantly than that in NCI-H1975 cells. The IC50 of paclitaxel for NCI-H1299 and NCI-H1975 cells was 101.4 nM and 22.61 nM, respectively (Fig. 4B), which suggested the induction of autophagy inversely correlated with the sensitivity of cells to drugs. Therefore, by blocking autophagic flux, hederagenin had a more significant sensitization effect on cancer cells with high levels of autophagy. To further support the notion that autophagy inhibition is the relevant mechanism underlying the synergy of hederagenin with chemotherapy, we used siATG5 to abolish autophagy induction before drugs treatment. As shown in Fig. S3, while ATG5 knockdown (hence autophagy-deficient) was sufficient to sensitize cytotoxicity of paclitaxel, addition of hederagenin did not further enhance the synergistic effects, indicating that modulation of autophagic flux is critical for hederagenin mediated actions.

Autophagy actually has multiple functions on tumor development. In the initial stage of the tumor, activation of autophagy can inhibit tumorigenesis by relieving the oncogenic microenvironment including chronic tissue damage, inflammation, and genomic instability^24,25^. When tumors develop to an advanced stage, autophagy may play the opposite roles helping tumor cells withstand survival pressures such as local anemia (glucose deficiency) and hunger^26,27^. Therefore, to treat tumor patients by regulating autophagy, individualized therapy strategies should be considered for different stages of tumor development. As a matter of fact, most cancer patients were diagnosed to reach a rather developed stage, where inhibiting autophagy was expected to achieve favorable outcomes in the clinical cancer therapy. In this study, we used NCI-H1299 and NCI-H1975 cell lines, both of which are characterized by high metastasis and thus represent advanced lung cancer cells. It was frequently reported that cisplatin or paclitaxel treatment may induce autophagy in different cancer cell types, including lung cancer cells. In this regard, inhibition of autophagy by pharmacologic agents or small interfering RNA sensitized cancer cells to cisplatin or paclitaxel^28–32^. Meanwhile, a series of phytochemicals, such as andrographolide, liensinine, and ginsenoside, have been identified as natural autophagy inhibitors and were further demonstrated to efficiently reduce resistance to chemotherapeutic reagents including cisplatin, doxorubicin, 5-fluorouracil, which strengthen the idea that inhibiting autophagy is a promising strategy in the comprehensive cancer therapies^33–35^. However, up to now, only CQ and its derivative HCQ have been used for treating patients with malignant tumors^36^. Other autophagy inhibitors that act through similar or different mechanisms with CQ/HCQ may offer additional prospects to improve chemotherapy under this strategy. In addition to CQ, bafilomycin A1 represents another class of autophagy inhibitor by strongly inhibiting lysosomal V-ATPase. It is not surprising that bafilomycin A1 was also frequently applied as an adjuvant agent for sensitizing chemotherapy in the pre-clinical models^37^. However, since bafilomycin A1 alone renders considerable cytotoxicity^38^, there will be major concerns on serious adverse events or treatment withdrawal in the clinical settings in future. In this regard, Hederagenin, which inhibited autophagy and cell survival with discriminate doses (dose for autophagy inhibition « dose for cell death) holds the potential to enhance chemotherapy while provide no additional adverse events.

Both CQ and bafilomycin A1 block autophagic flux by preventing lysosomal fusion to autophagosome and inhibiting lysosomal acidification. Hederagenin, however, appear to act through distinct mechanisms. First of all, the analysis on GFP-LC3 and LysoBrite^TM^ red demonstrated that almost all the GFP-LC3 positive cellular structures were co-localized with lysosomes, which was in sharp contrast to the situation after CQ treatment (Fig. 2A). Meanwhile, under the transmission electron microscopy, we failed to identify autophagosome-like double membrane-bound vesicles in cells treated with hederagenin (Fig. 2F). Instead, single membrane vesicle containing cell organelles was the most evident cell ultrastructure (Fig. 2F). We concluded that unlike CQ and bafilomycin A1, hederagenin did not affect the formation of autolysosomes. On the other hand, although a series of evidences supported hederagenin might inhibit lysosomal acidification, this was not achieved through interfering with V-ATPase activity, as bafilomycin A1 did (Fig. S4). At the concentration of autophagy inhibition, hederagenin failed to reduce any enzyme activity of V-ATPase (Fig. S4). Therefore, other lysosomal proteins (e.g., MCOLN3)^39^, which are also involved in the maintenance of lysosomal pH, could be potential targets of hederagenin. Further investigations are required to identify the molecular targets of hederagenin. Nonetheless, it is sufficient to conclude that hederagenin could disturb late-stage autophagy by raising the lysosomal pH. Thus, hederagenin that has a high inhibitory effect on autophagy but with low cytotoxicity is expected to be an ideal sensitization adjuvant of chemotherapy for lung cancer.

The synergistic killing action of hederagenin combined with cisplatin or paclitaxel on lung cancer cells might result from the elevated intracellular ROS levels which can directly activate autophagy^40,41^. For instance, it was reported that lysosomal calcium channel MCOLN1 could sense mitochondria-derived ROS and lead to calcium release. This in turn induced calcineurin-dependent TFEB activation for autophagy induction^42^. On the other hand, autophagy is well-known to eliminate intracellular ROS. For example, mitophagy induction may lead to degradation of damaged mitochondria, which are the source of ROS. In this way, mitophagy is thought to protect cells from ROS-mediated death and confer drug resistance to cancer cells^43,44^. In the present study, we demonstrated that paclitaxel and cisplatin-induced ROS production alongside autophagy induction in lung cancer cells (Fig. 5B-D). This observation was consistent with previous reports that these anticancer drugs may cause ROS generation that participates in autophagy induction^21,22^. Interestingly, autophagy inhibition by hederagenin caused significantly higher ROS levels in cells treated with paclitaxel or cisplatin. This finding supported the notion that autophagy is crucial for ROS elimination. Accompanied by elevated ROS, combination of hederagenin with paclitaxel or cisplatin presented significantly higher cytotoxicity to cancer cells than individual agent alone (Fig. 3A-B and Fig. 4D-E). To corroborate the role of ROS, their scavenging by NAC abolished this sensitizing effect of hederagenin (Fig. 5E-G).

Tandem fluorescent tagged (i.e., mCherry and GFP) LC3 is a sensitive assay that can be adopted to detect aberrant pH environment in acidic organelle (i.e., autolysosome) due to the treatment of various drugs^45^. Such assay has been exploited for high throughput screening of novel inhibitors of autophagic flux^46^. The pKa of GFP, which reflects the ratio of deprotonated GFP (absorb light at 488 nm and emits light peaking at 508 nm) and protonated GFP (no absorbance of light) is around 6.0, indicating that at pH 6.0, only 50% of the GFP are at protonated state and hence emit no light. Analysis of the relative fluorescence (RFU)-pH relationship revealed a double-exponential model for GFP, demonstrating that the RFU drops sharply at pH ranging from 6 to 4^47^. These analyses implied that even mild pH changes in this pH range would greatly modify the fluorescent intensity of GFP. For example, the RFU of GFP at pH 6 is 50%, while it drops to <10% at pH 5.0. The elevation of pH by compounds treatment (Fig. 2C), i.e., hederagenin (pH 5.6) and bafilomycin A1 (pH 6.07) was sufficient to cause evident increase of GFP fluorescence, compared with vehicle (pH 4.97). In addition, GFP can be prevented for degradation by lysosomal hydrolases^45^, the activation of which required maturation yet had been suppressed by hederagenin (Fig. 2D, E), may further contribute to increase of GFP fluorescence. We therefore concluded that increased overlap of GFP and mCherry signals represented a compelling evidence that hederagenin may interfere with acidification of lysosomes and autolysosomes.

Given that hederagenin could alter pH in lysosomes/autolysosomes, this raised the possibility that hederagenin might act as a novel lysosomotropic agent. Previous studies reported that lysosomotropic agents, such as azadirachtin and staurosporine, may increase lysosomal membrane permeabilization (LMP)^48,49^, which may cause the leakage of hydrogen ions from lysosomes and result in the elevation of lysosomal pH eventually^50^. Meanwhile, increased LMP may also lead to the release of lysosomal enzymes (such as cathepsin B and cathepsin D) into the cytoplasm, contributing to lysosomal apoptosis^51^. Our data showed that hederagenin has no notable cytotoxicity at the concentration of autophagy inhibition, suggesting that hederagenin doesn’t appear to act through increasing LMP.

In summary, hederagenin inhibits lysosome acidification and thereby impairing autophagic flux in lung cancer cells. Such effects lead to failure in ROS clearance, which in turn enhanced the cell death caused by paclitaxel and cisplatin. In this capacity, hederagenin represents a novel candidate as an adjuvant for enhancing the antineoplastic effects of conventional chemotherapeutics. It is hopeful that further clinical studies in the future could demonstrate the use of hederagenin for improving the efficacy of chemotherapy or alleviating treatment-related side effects by reducing the doses of anticancer agents.

## Conclusions

In our study, we provide extensive evidence that hederagenin acted as a novel late-phase autophagy inhibitor by targeting lysosomes, and that hederagenin may synergize the cytotoxicity effects of cisplatin and paclitaxel by blocking the cell death preventive autophagy and amplifying cytotoxic ROS. We believe our finding holds scientific merits regarding the knowledge and translation of autophagy modulators.

## Supplementary information

Supplementary Figure Legends

Figure S1

Figure S2

Figure S3

Figure S4
