# Supplementary material for: Hederagenin potentiated cisplatin- and paclitaxel-mediated cytotoxicity by impairing autophagy in lung cancer cells
Source: Cell Death Dis. 2020 Aug 13;11(8):611. doi: 10.1038/s41419-020-02880-5 (PMC7426971; doi:10.1038/s41419-020-02880-5)
Supplement: Supplementary file 1 — Supplementary Figure Legends [file 41419_2020_2880_MOESM1_ESM.docx]

**Supplementary Figure Legends**

**Fig. S1. Hederagenin inhibited autophagy flux in several kinds of cancer cells.** Cells were treated by hederagenin (25, 50, 75 μM) or vehicle for 24h, then the indicated proteins were detected by western blot assay. The data showed hederagenin caused p62 accumulation and LC3-II upregulation in a dose-dependent manner. Hederagenin: Hed

**Fig. S2. Cells were stained with AO.** Low pH-dependent red fluorescence was abolished after hederagenin and bafilomycin A1 treatment. Scale bar, 20 μm.

**Fig. S3 Synergistic effects of hederagenin were diminished in the autophagy-deficient cell model (ATG5 knockdown by siRNA transfection). (A)** NCI-H1299 cells were transfected by negative-control and ATG5 siRNAs for 36h, then the total protein was harvested. ATG5 expression was determined by western blot assay, β-actin was used as an internal reference. The intensity of the bands was measured by Gel-Pro software. NC, negative control. **(B)** NCI-H1299 cells were seeded into 96-well plates, then cells were transfected by ATG5 siRNA for 24h followed by drug treatment as indicated for another 24h. The cell viability was determined by CCK8 assay. ns, no significance, ***p < 0.001, ANOVA with multiple comparisons. Paclitaxel: PTX, hederagenin: Hed.

**Fig. S4. The effects of hederagenin on lysosomal V-ATPase.** Compared with DMSO (Ctrl), bafilomycin A1 (Baf, positive control) significantly reduced V-ATPase activity in crude lysosomes. There was no difference in V-ATPase activity between Hederagenin (Hed) and DMSO treated lysosomes. ns, no significance, *p < 0.05, ANOVA with multiple comparisons. V-ATPase assay kit (Cat NO. GMS50244) was purchased from Genmed Scientifics (Arlington, MA, USA). The effects of hederagenin or bafilomycin A1 on V-ATPase activity were determined according to the manufacturer’s instructions. Lysosomes were enriched from untreated NCI-H1299 cells and aliquoted into three groups. The lysosomes in each group were further aliquoted into two separated tubes (for total and unspecific ATPase activity detection), mixed with all the reagents provided by the kit (Reagent A-H), and then treated with DMSO (Ctrl), hederagenin (Hed) or bafilomycin A1 (Baf), respectively. The mixtures from each tube were recorded under 340 nm immediately, and then recorded again after 10 minutes. All the recording were performed at 37 °C. The activity of V-ATPase was calculated by subtracting unspecific ATPase activity from total ATPase activity. The activity from each group were normalized with DMSO (Ctrl) group and compared using ANOVA with multiple comparisons.
