# Supplementary figures and images for: Hederagenin potentiated cisplatin- and paclitaxel-mediated cytotoxicity by impairing autophagy in lung cancer cells
Source: Cell Death Dis. 2020 Aug 13;11(8):611. doi: 10.1038/s41419-020-02880-5 (PMC7426971; doi:10.1038/s41419-020-02880-5)

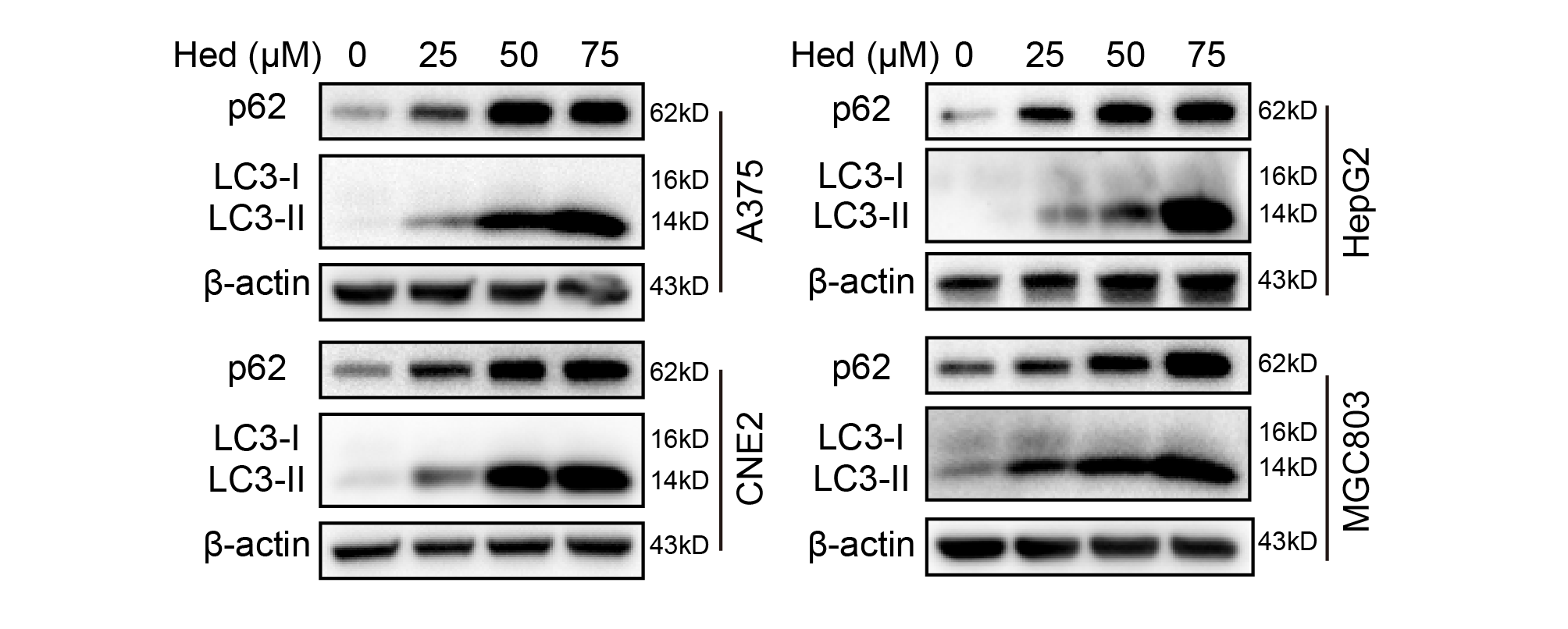

Supplement: Supplementary file 2 — Figure S1 [file 41419_2020_2880_MOESM2_ESM.png]

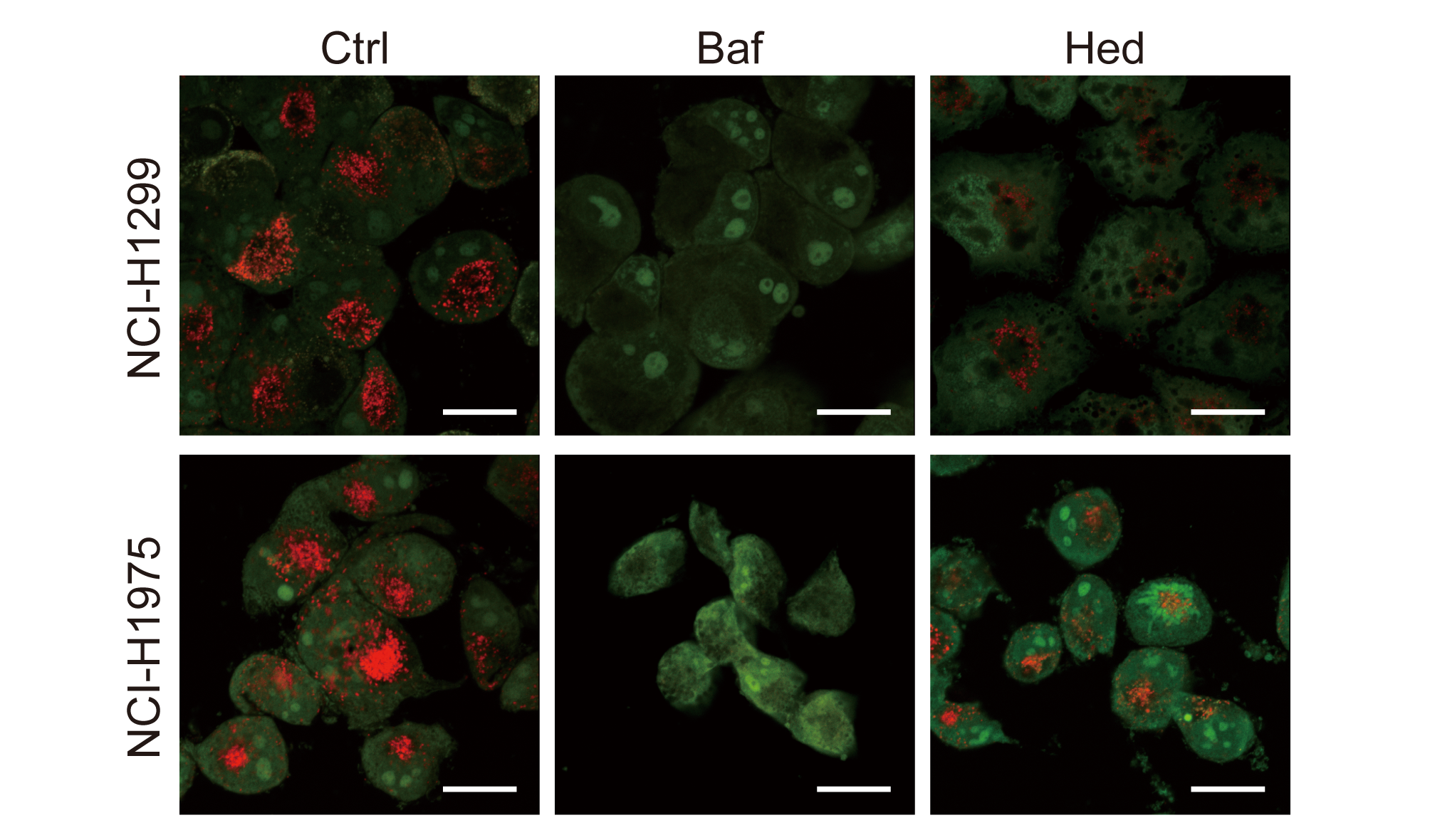

Supplement: Supplementary file 3 — Figure S2 [file 41419_2020_2880_MOESM3_ESM.png]

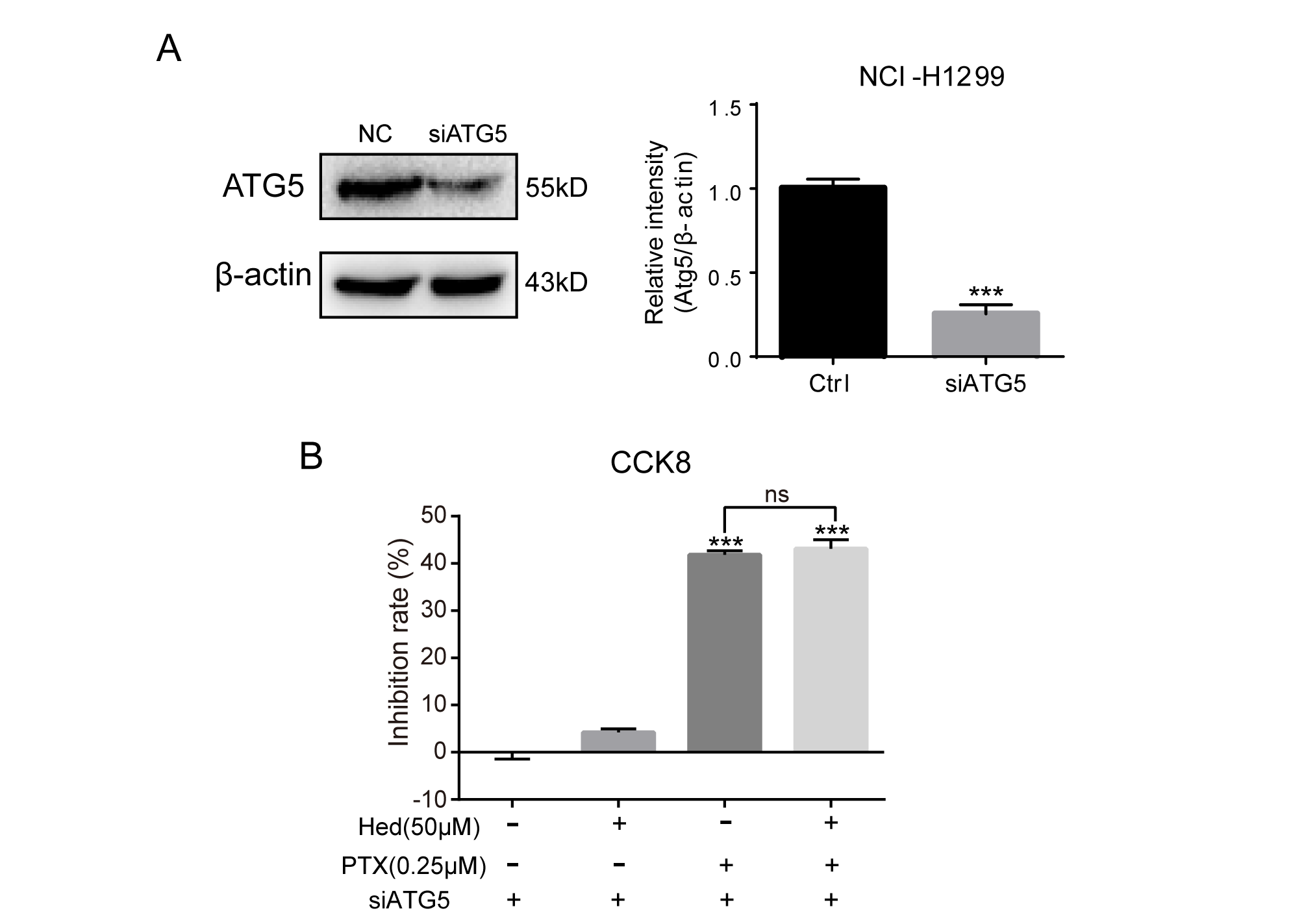

Supplement: Supplementary file 4 — Figure S3 [file 41419_2020_2880_MOESM4_ESM.png]

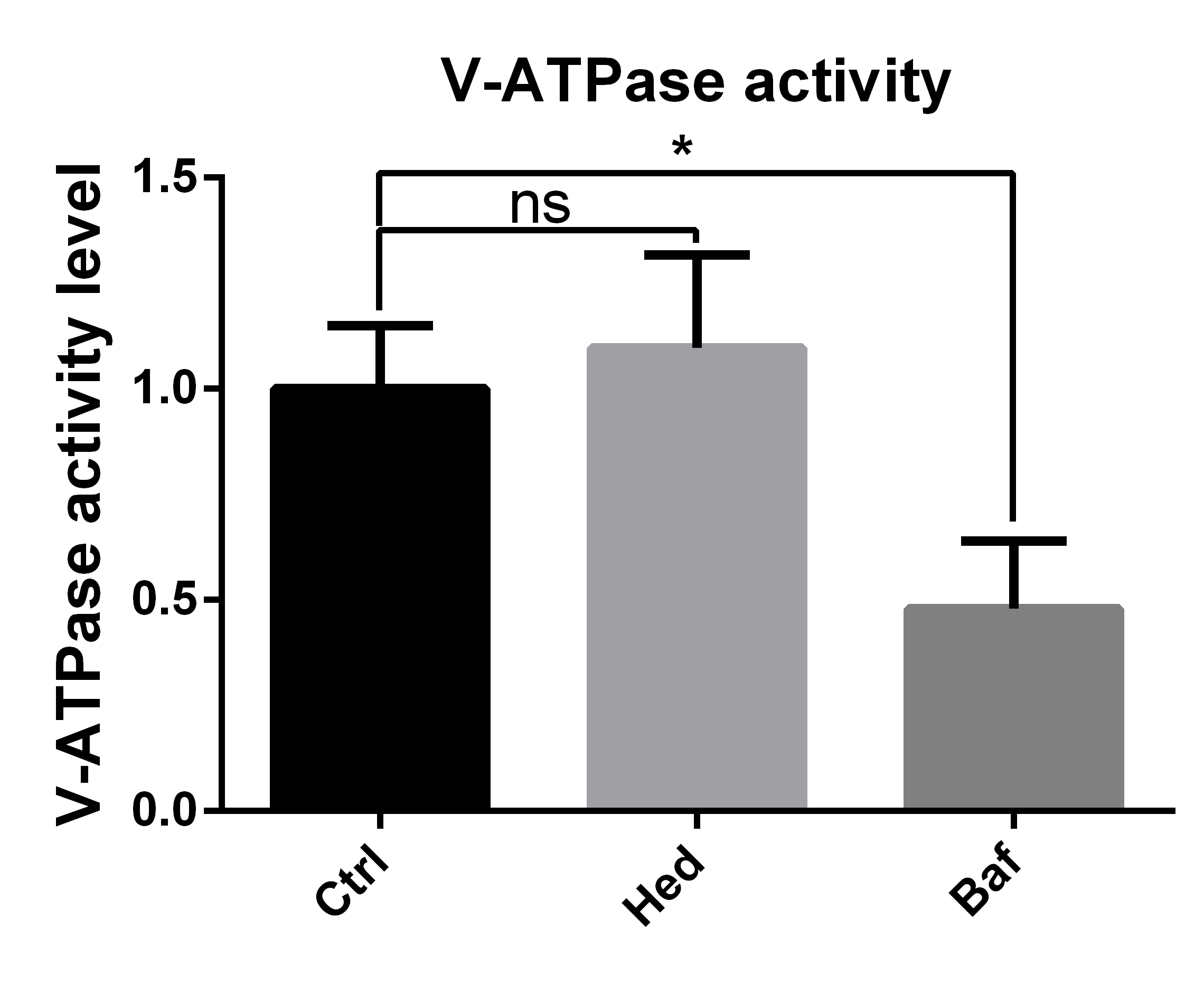

Supplement: Supplementary file 5 — Figure S4 [file 41419_2020_2880_MOESM5_ESM.png]
